# The Biosynthesis Pattern and Transcriptome Analysis of *Sapindus saponaria* Oil

**DOI:** 10.3390/plants13131781

**Published:** 2024-06-27

**Authors:** Xiao Zhou, Lijuan Jiang, Peiwang Li, Jingzhen Chen, Yunzhu Chen, Yan Yang, Luhong Zhang, Yuena Ji, Zhihong Xiao, Kezhai Sheng, Xiaoqian Sheng, Hui Yao, Qiang Liu, Changzhu Li

**Affiliations:** 1 College of Life and Environmental Sciences, Central South University of Forestry and Technology, Changsha 410004, China; zx924647234@163.com (X.Z.); znljiang2542@163.com (L.J.); 20210100019@csuft.edu.cn (L.Z.); 2 State Key Laboratory of Utilization of Woody Oil Resource, Hunan Academy of Forestry, Changsha 410018, China; lindan523@163.com (P.L.); chenjingzhen621@sina.com (J.C.); cyzcarol@foxmail.com (Y.C.); yangyanzupei@126.com (Y.Y.); jyn13875854782@163.com (Y.J.); xzhh1015@163.com (Z.X.); 3 Key Laboratory of National Forestry and Grassland Administration on Utilization Science for Southern Woody Oilseed, Hunan Academy of Forestry, Changsha 410018, China; 4 Hunan Soapberry Agroforestry Development Co., Ltd., Changde 415325, China; 18207366789@163.com; 5 Shimen County Forestry Bureau, Changde 415300, China; 13203640193@163.com (X.S.); yaohui1234@163.com (H.Y.)

**Keywords:** *Sapindus saponaria*, triacylglycerol, fatty acid, woody oil plant, lipid metabolism, transcriptome analysis, oil biosynthesis, key enzyme genes, biodiesel, soapberry saponin

## Abstract

The *Sapindus saponaria* (soapberry) kernel is rich in oil that has antibacterial, anti-inflammatory, and antioxidant properties, promotes cell proliferation, cell migration, and stimulates skin wound-healing effects. *S. saponaria* oil has excellent lubricating properties and is a high-quality raw material for biodiesel and premium lubricants, showing great potential in industrial and medical applications. Metabolite and transcriptome analysis revealed patterns of oil accumulation and composition and differentially expressed genes (DEGs) during seed development. Morphological observations of soapberry fruits at different developmental stages were conducted, and the oil content and fatty acid composition of the kernels were determined. Transcriptome sequencing was performed on kernels at 70, 100, and 130 days after flowering (DAF). The oil content of soapberry kernels was lowest at 60 DAF (5%) and peaked at 130 DAF (31%). Following soapberry fruit-ripening, the primary fatty acids in the kernels were C18:1 (oleic acid) and C18:3 (linolenic acid), accounting for an average proportion of 62% and 18%, respectively. The average contents of unsaturated fatty acids and saturated fatty acids in the kernel were 86% and 14%, respectively. Through the dynamic changes in fatty acid composition and DEGs analysis of soapberry kernels, *FATA*, *KCR1*, *ECR*, *FAD2* and *FAD3* were identified as candidate genes contributing to a high proportion of C18:1 and C18:3, while *DGAT3* emerged as a key candidate gene for TAG biosynthesis. The combined analysis of transcriptome and metabolism unveiled the molecular mechanism of oil accumulation, leading to the creation of a metabolic pathway pattern diagram for oil biosynthesis in *S. saponaria* kernels. The study of soapberry fruit development, kernel oil accumulation, and the molecular mechanism of oil biosynthesis holds great significance in increasing oil yield and improving oil quality.

## 1. Introduction

The soapberry, scientifically known as *Sapindus saponaria* and belonging to the deciduous tree family Sapindaceae, thrives in the eastern and southern regions of China, with its distribution extending gracefully towards the southwestern territory. Moreover, this multifunctional plant can be discovered in Japan, North Korea, the Indochina Peninsula, and India [1]. The wide ecological range and remarkable adaptability of *S. saponaria* endow it with resistance to drought and barren conditions, rendering it a prominent woody oil plant for effectively combating ecological rocky desertification [2]. The soapberry is widely acclaimed for its high economic value, owing to the precious fruit oil and saponin it possesses [3]. The kernel has an oil content that can reach as high as 42.7% (*w*/*w*), and its oil has many high-quality characteristics, such as a high content of unsaturated fatty acids (UFA) and excellent lubricating properties, making it a high-quality raw material for the production of biodiesel [1,4]. Current research on *S. saponaria* oil primarily focuses on the determination of its oil content [4], analysis of its oil characteristics [5], optimization of extraction processes [6], preparation of biodiesel [5], and increasing its application in other areas, such as anti-inflammatory, antibacterial, and antioxidant effects [7,8,9]. In vivo experiments have shown that *S. saponaria* oil can accelerate skin wound-healing and has anti-inflammatory, antibacterial, and antioxidant effects, making it suitable for developing drugs to treat skin injuries [7]. Its oil can enhance cell migration ability and promote cell proliferation, as well as promote the proliferation, osteogenic differentiation, and secretion of matrix vesicles of human dental pulp mesenchymal stem cells [7,8,9]. It can be used as a material for the prevention of oral diseases. In recent years, there has been an increasing amount of development and utilization of soapberry oil, but there is limited research on elucidating the biological synthesis mechanism underlying its oil.

The triacylglycerol (TAG) represents the predominant form of plant oil found in plant fruits [10]. The biological synthesis pathway of lipids is a highly intricate process that can be categorized into three distinct stages, as follows: fatty acid (FA) synthesis, acyl-based elongation and modification, and TAG assembly. These processes primarily take place within the cytosol, mitochondria, endoplasmic reticulum (ER), and oil cells, involving numerous pivotal enzymes and regulators [11]. The diversity of plant oils stems from the different gene expression patterns of different plants. The activity of key enzymes in each stage of oil synthesis will play a role, whether high or low, which leads to the uniqueness of its oil. De novo synthesis, acyl extension, and editing of FAs determine the composition and content differences of FAs, while the assembly of TAGs is key to determining the differences in oil content. FA de novo synthesis and acyl extension occur in the plastids. FA de novo synthesis involves the synthesis of butyryl acid carrier protein (ACP), using acetyl coenzyme (CoA) and malonyl-CoA as substrates, through condensation, reduction, dehydration, and re-reduction under the catalysis of the FA synthase complex [11]. FA acyl extension and editing are a series of reactions catalyzed by the FA synthase complex, using butyryl-ACP instead of acetyl-ACP and malonyl-ACP as substrates. Under the catalysis of the FA synthase complex, they undergo condensation, reduction, dehydration, and re-reduction again to form hexanoyl-ACP (C6:0-ACP). By analogy, C6:0-ACP, octanoyl-ACP (C8:0-ACP), decanoyl-ACP (C10:0-ACP), dodecanoyl-ACP (C12:0-ACP), and tetradecanoyl-ACP (C14:0-ACP) serve as substrates for the next cycle of FA synthase complex catalysis, ultimately forming palmitoyl-ACP (C16:0-ACP). The elongation of the carbon chain of C16:0-ACP is catalyzed by KASII (3-oxoacyl-ACP synthase II), leading to the formation of C18:0-ACP. Subsequently, SAD (acyl-ACP desaturase) desaturates this intermediate to produce C18:1-ACP. Fatty acyl-ACP thioesterase hydrolyzes the acyl-ACP produced from these processes to form corresponding free fatty acids (FFAs) [11,12]. These FFAs need to be converted into fatty acyl CoA by long-chain acyl-CoA synthetase (LACS) for FA elongation or participation in glycerolipid metabolism. TAG assembly occurs in the ER, where various acyltransferases catalyze the connection of three molecules of FAs and glycerol through ester bonds to form TAG.

The high content of C18:1 in *Symplocos paniculata* [13] is related to its high expression of KASII. The high content of C18:2 (linoleic acid) in walnuts (*Juglans regia*) is related to its high expression of SAD, FAD2 (ω-6 FA desaturase) and FAD3 (ω-3 FA desaturase) [14], and the high content of C18:3 in flax (*Linum usitatissimum*) is related to its high expression of FAD2 and FAD3 [15]. By over-expressing *LuFAD2* and *LuFAD*3 in *Arabidopsis* seeds, it was found that their FA content significantly increased, especially in PUFA (polyunsaturated fatty acid) content [16]. *KASII*, *SAD*, *FAD2* and *FAD3* can serve as regulatory candidate genes for high levels of C18:1, C18:2, and C18:3. There are also studies indicating that the interaction between *DGAT1* (diacylglycerol O-acyltransferase 1) and *PDAT1* (phospholipid: diacylglycerol acyltransferase 1) can enhance the assembly of *Arabidopsis* TAG [17]. Improving enzyme activity during TAG assembly can accelerate oil accumulation. The transcriptomics and metabolomics analysis of woody oil plants, such as *Prunus pedunculata* [18], *Prunus sibirica* [19], and *Vernicia fordii* [20], have shed light on the intricate mechanisms underlying their oil biosynthesis and accumulation patterns. However, the elucidation of the oil biological synthesis and accumulation mechanism in *S. saponaria* remains unexplored. To unravel the distinctive FA composition in *S. saponaria*, a comprehensive analysis of transcriptome and metabolomics was conducted on kernels to unveil the different expression patterns of genes implicated in oil biosynthesis. In this investigation, we scrutinized the morphological features of *S. saponaria* fruits at diverse stages from 0 to160 days after flowering (DAF) and ascertained the kernel oil content and FA composition. Based on these observations, RNA-Seq was executed during three pivotal phases of kernel development and oil accumulation in the soapberry fruit. The study of oil accumulation patterns and biosynthetic molecular mechanisms in *S. saponaria* can offer theoretical guidance for enhancing its oil production and quality.

## 2. Results

### 2.1. Dynamic Patterns of Fruit Oil Changes and Development

**Figure 1:**
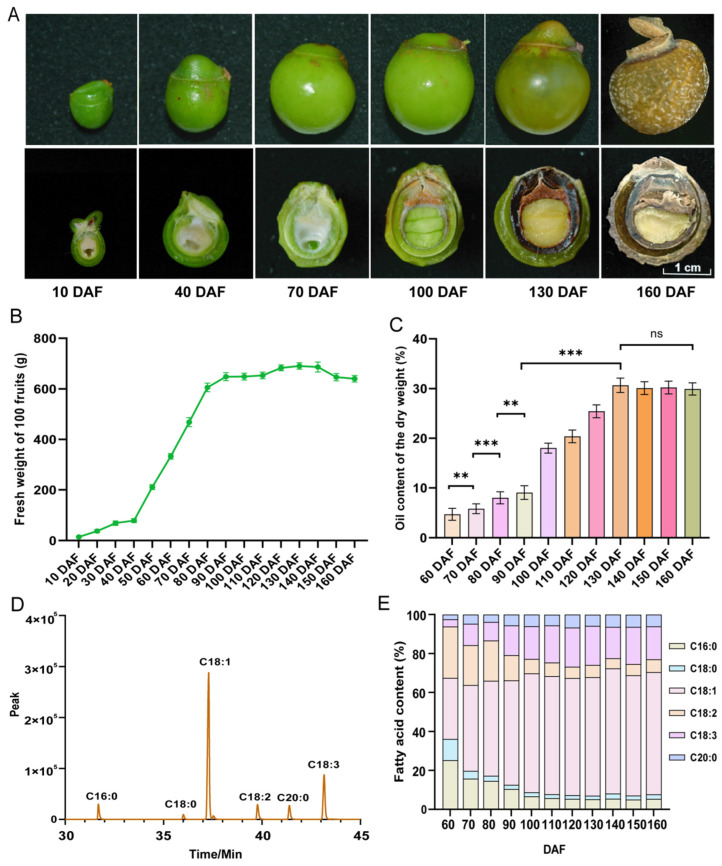
Phenotypic characteristics, oil content, and fatty acid dynamics of *S. saponaria* fruit at different developmental stages: (**A**) phenotypic characteristics of *S. saponaria* fruits; (**B**) changes in fresh weight of 100 fruits of *S. saponaria*; (**C**) changes in oil content in *S. saponaria* kernels; (**D**) the gas chromatography peak plot of *S. saponaria* kernel oil in 130 DAF; and (**E**) the fatty acid content of *S. saponaria* kernels. Note: ** means oil content with significant difference, *** means oil content with very significant difference, ns means oil content with insignificant differences. C18:1: oleic acid, C18:3: linolenic acid, C18:2: linoleic acid, C20:0: arachidic acid, C16:0: palmitic acid, C18:0: stearic acid.

The phenological observation revealed that the young fruit stage of *S. saponaria* commences at 10 DAF (mid-June). Based on the fluctuations in the fresh weight of 100 soapberry fruits, fruit development can be roughly categorized into three stages. In the initial stage (0–40 DAF), there is a gradual increase in fruit weight, ranging from 15 g to 78 g. Subsequently, there is a consistent linear growth phase (40–90 DAF), with the weight progressively rising from 78 g to 648 g. Finally, the fruit enters a stable growth period (90–160 DAF), reaching its maximum weight of 690 g at 130 DAF (Figure 1B).

The trend of oil content exhibited a consistent pattern with the fresh weight of 100 soapberry fruits. During the initial developmental stage of soapberry kernels (0–50 DAF), which exist in the form of liquid endosperm, no detectable oil was observed (Figure 1A). The kernels undergo a slow accumulation stage of oil content between 60–90 DAF, during which the oil content increases from 5% to 9%. Subsequently, a rapid accumulation stage occurs between 90–130 DAF, leading to an increase in oil content from 9% to 31%. The oil content of the kernels remains stable level in the 130–160 DAF, exhibiting no significant changes and reaching a maximum value of 31% oil content at 130 DAF (Figure 1C). Based on the dynamic pattern of soapberry kernel oil, kernel development can be categorized into three stages, as follows: a period of slow oil accumulation (60–90 DAF), a period of rapid oil accumulation (90–130 DAF), and a period of stable oil accumulation (130–160 DAF). For the subsequent RNA-Seq analysis, representative kernels were selected from each developmental stage: 70 DAF (S1), 100 DAF (S2), and 130 DAF (S3).

### 2.2. Dynamic Content of Fatty Acids

Using gas chromatography (GC), the content of C18:1, C18:2, C18:3, C20:0, C16:0, and C18:0 was determined (Figure 1C,E). From the perspective of dynamic changes in FA percentage, significant changes were observed between 60–100 DAF, and the FA content tended to stabilize after 130 DAF. The content of C18:1, C18:3, and C20:0 showed an upward trend throughout the entire kernel development process, while C18:2, C16:0, and C18:0 exhibited a decreasing trend. During 60–100 DAF, C18:1, C18:2, and C16:0 account for the large proportion in all FA, with average proportions of 48%, 18%, and 15%, respectively, while C18:3, C20:0, and C18:0 account for a smaller proportion, with average proportions of 11%, 5%, and 4%, respectively. At 100–160 DAF, among the six FAs, C18:1 accounted for the largest proportion, with an average of 62%; followed by C18:3, with an average content of 18%; while the average proportions of C18:2, C20:0, C16:0, and C18:0 after 100 DAF were 6%, 6%, 6%, and 2%, respectively. The *S. saponaria* kernel FAs from 60 to 130 DAF showed the following changes: C18:1 increased from 31% to 61%; C18:3 content increased from 4% to 20%; C20:0 increased from the initial 2% to 6% at 130 DAF (Figure 1E). The percentage of UFAs showed an increasing trend, rising from 61% (60 DAF) to 86% (130 DAF); while the percentage of saturated fatty acids (SFAs) decreased from 39% (60 DAF) to 14% (130 DAF). After soapberry fruit ripening (130–160 DAF), the average percentage of UFAs and SFAs in the kernel was 86% and 14%, respectively.

### 2.3. RNA-Seq and Sequence Alignment

To elucidate the genetic expression changes and underlying molecular mechanisms governing soapberry fruit development, we performed RNA-Seq analysis at three pivotal stages of oil accumulation in *S. saponaria* kernels. Following sequencing, the transcriptome RawDatas were subjected to stringent filtering, resulting in 407,463,420 (60,716,969,523 bp) high-quality CleanData (Clean reads) out of 410,003,474 (61,500,521,100 bp) RawDatas. This accounted for an 99.38% of the total dataset. The filtered CleanData exhibited remarkable characteristics with average percentages of Adapters and LowQuality at a mere 0.05% and 0.57%, respectively. Additionally, the GC content was found to be significantly enriched at 45.91%. Furthermore, quality scores Q20 (%), Q30 (%) reached high levels of accuracy at 97.51% and 93.01%, respectively (Table 1). Finally, the presence of polyA sequences in the filtered CleanData was negligible at only 0.00%. Moreover, the percentage of N bases in the CleanData was less than a minimal threshold value of <0.01%.

By comparing all CleanData with 30,068 genes in the *S. saponaria* genome, a total of 20,146 genes were compared across nine soapberry kernels from three periods, resulting in a comparison rate of 67.00%. Specifically, during the sequence alignment process, novel genes that were not included in the reference genome were discovered and reconstructed using Stringtie to generate transcripts, which were then annotated. A total of 3391 new genes were identified, with S1 having 2810 genes; S2 having 1959 genes; and S3 having 1691 genes.

### 2.4. Transcriptome Annotation and Gene Expression Profiling

The comparison of all CleanData with the NCBI non-redundant protein database (NR) revealed a total of 28,221 single genes that were successfully annotated, resulting in an annotation rate of 84.35%. Furthermore, when compared to the Gene Ontology (GO) database, it was observed that the Cellular Component (CC) category exhibited the highest number of annotations among the GO functional categories, including Molecular Function (MF) and Biological Process (BP), as depicted in Figure S1A. The clean reads were simultaneously annotated using the KEGG pathway database. The annotation results revealed a total of 9625 annotated genes. Within the KEGG A class, there were 5 major categories, while the KEGG B class comprised 19 subcategories (Figure S1B). Notably, ‘Metabolism’ exhibited the highest gene count in the A-level classification, with 5501 genes, accounting for 57.15%. Furthermore, it encompassed 11 B-level subcategories. The KEGG A-level classification revealed that the categories of Transport and Catabolism, as well as Environmental Adaptation, exhibited the smallest gene counts, with 286 and 258 genes, respectively. Notably, each category solely encompasses one subcategory within the KEGG B-level classification. Among a total of 28,221 individual genes examined, it is noteworthy that 372 genes are implicated in lipid metabolism.

### 2.5. Differential Gene Expression Analysis

**Figure 2:**
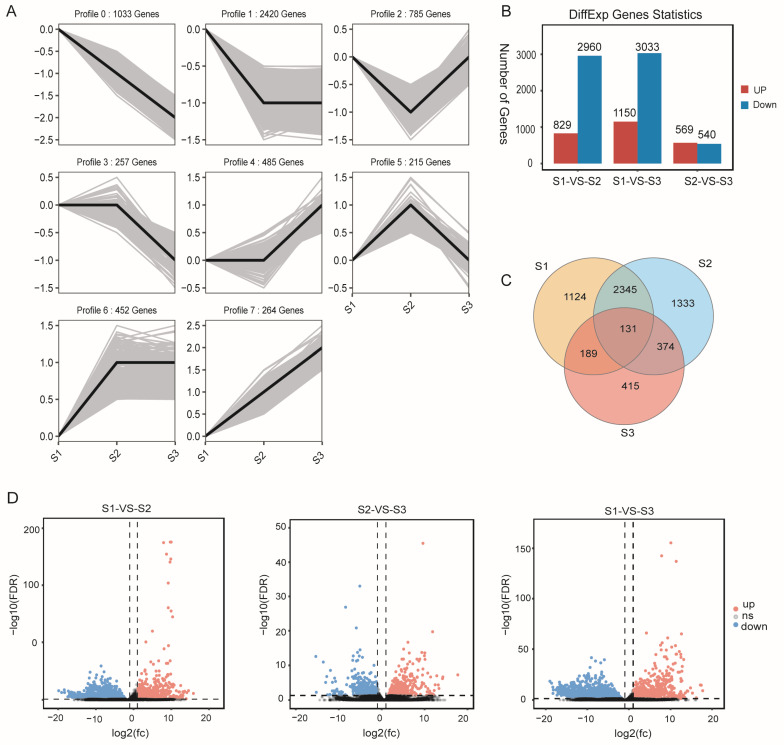
Analysis of DEGs in the transcriptome of *S. saponaria* kernels: (**A**) trend of DEGs in *S. saponaria* kernels transcriptome; (**B**) statistics of up-regulation and down-regulation of DEGs in the transcriptome of *S. saponaria* kernels; (**C**) Venn diagram of DEGs in the transcriptome of *S. saponaria* kernels; and (**D**) volcano plots showing up-regulation and down-regulation of DEGs in *S. saponaria* kernels. Note: grey represents the actual FPKM of DEGs, while black represents the average FPKM value in (**A**); up means significantly up-regulated genes, down means significantly down-regulated genes, and ns (dotted line) means genes with insignificant differences in (**D**).

The expression levels of genes in each sample were quantified using RSEM. The gene expression data of the soapberry kernel transcriptome were normalized using the fragments per kilobase of exon model per million mapped fragments (FPKM) method. The DESeq2 algorithm was employed to identify DEGs in soapberry kernels, with a stringent threshold of |log2(FC)| > 1 and FDR < 0.05. As a result, a total of 5911 DEGs were successfully identified (Figure 2B,C). By conducting trend analysis on the FPKM values of these DEGs, it was observed that they could be classified into eight distinct profiles, with profile 0 and profile 1 exhibiting the highest number of DEGs (Figure 2A). The majority of DEGs in the kernels displayed a down-regulated expression pattern, which was consistent with the overall gene expression trend observed throughout the three time periods. In the comparison between S1 and S2, 829 genes exhibited up-regulation, whereas 2960 genes displayed down-regulation. In the comparison between S1 and S3, 1150 genes were found to be up-regulated, while 3033 genes showed down-regulation. Regarding the comparison between S2 and S3, there was an up-regulation of 569 genes and a down-regulation of 540 genes. Among the DEGs, a total of 1124 genes exhibited unique expression in S1, while 1333 genes were uniquely expressed in S2, and 415 genes showed exclusive expression in S3. Furthermore, there was a subset of 131 co-expressed genes that demonstrated consistent expression across all three time points (Figure 2D).

**Figure 3:**
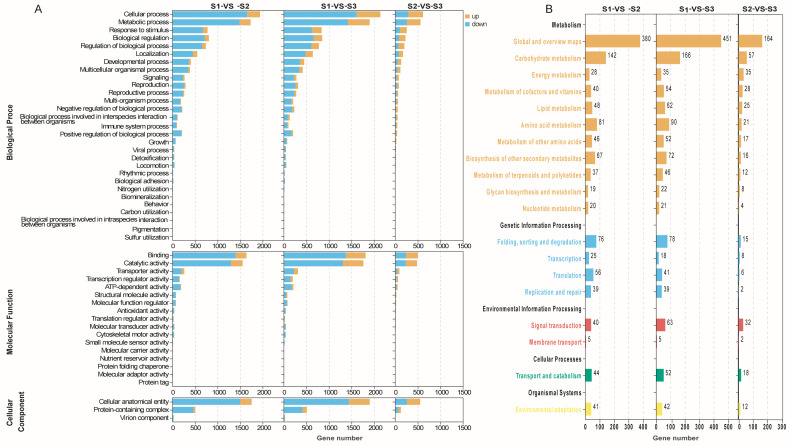
GO and KEGG annotation of DEGs in the transcriptome of *S. saponaria* kernels: (**A**) annotation on GO functional classification of DEGs in *S. saponaria* kernels; and (**B**) enrichment of KEGG metabolic pathway of DEGs in *S. saponaria* kernels.

The DEGs obtained from the comparisons between S1 and S2, S2 and S3, and S1 and S3 were subjected to functional annotation using Gene Ontology (GO) analysis and metabolic pathway enrichment analysis based on the Kyoto Encyclopedia of Genes and Genomes (KEGG) database (Figure 3A,B). In the BP category, cellular process and metabolic process exhibited the highest number of up-regulated genes as well as down-regulated genes. In MF, binding and catalytic activity demonstrated a significant abundance of both up-regulated and down-regulated genes, whereas in CC, cellular anatomical entity displayed the largest count of up-regulated genes and down-regulated genes (Figure 3A). Similarly, the KEGG metabolic pathway enrichment analysis classified all DEGs into five A-level categories and 19 B-level subcategories, encompassing a total of 133 metabolic pathways (Figure 3B). The numbers of KEGG enriched metabolic pathways in the S1-vs-S2, S1-vs-S3, and S2-vs-S3 comparisons were 126, 131, and 107, respectively. These pathways involved a total of 1234, 1409, and 482 genes, respectively, which aligns with the quantitative trend observed for their DEG. Specifically, the number of enriched pathways and genes followed a pattern of S1-VS-S3 > S1-VS-S2 > S2-VS-S3. Among all the genes, 372 genes were annotated in lipid metabolism, including 337 DEGs. Of these DEGs, comparisons of S1-VS-S2, S1-VS-S3, and S2-VS-S3 were enriched with 48, 62, and 25 DEGs, respectively, in lipid metabolism.

### 2.6. Analysis of Lipid Metabolism DEGs

Through KEGG metabolic pathway enrichment of all DEGs in the transcriptome of *S. saponaria* kernel, a total of 13 lipid metabolism pathways were found to be significantly enriched (Table S3). These pathways primarily include the biosynthesis of unsaturated FAs (ko01040), glycerophospholipid metabolism (ko00564), glycerol metabolism (ko00561), FA elongation (ko00062), FA biosynthesis (ko00061). The S1-VS-S2, S2-VS-S3, and S1-VS-S3 comparisons revealed enrichment of 12, 11, and 13 metabolic pathways, respectively. These comparisons involved a total of 67, 30, and 87 DEGs, with 21 up-regulated genes and 46 down-regulated genes in the S1-VS-S2 comparison; 9 up-regulated genes and 21 down-regulated genes in the S2-VS-S3 comparison; and finally, 35 up-regulated genes and 52 down-regulated genes in the S1-VS-S3 comparison.

### 2.7. Identification and Expression Profiles of Key DEGs for FA Biosynthesis, Elongation and Desaturation

**Figure 4:**
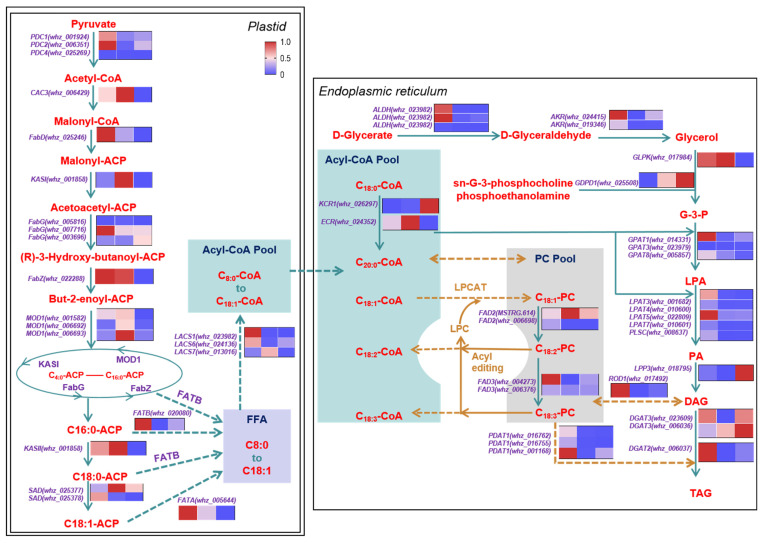
Schematic diagram of the biosynthetic metabolic pathway of FA and TAG in *S. saponaria* kernels. Note: pyruvate dehydrogenase complex: PDC; acetyl-CoA carboxylase: CAC3; ACP-S-malonyltransferase: FabD; 3-oxoacyl-ACP synthase: KAS; 3-oxoacyl-ACP reductase: FabG; 3-hydroxyacyl-ACP dehydratase: FabZ; enoyl-ACP reductase I: FabI, MOD1; acyl-ACP desaturase: SAD; fatty acyl-ACP thioesterase A: FATA; fatty acyl-ACP thioesterase B: FATB; long-chain acyl-CoA synthetase: LACS; very-long-chain 3-oxoacyl-CoA reductase: ECR; very-long-chain enoyl-CoA reductase: KCR1; ω-6 FA desaturase: FAD2; ω-3 FA desaturase: FAD3; acetaldehyde dehydrogenase: ALDH; alcohol dehydrogenase: AKR; glycerol kinase (ATP: glycerol-3-phosphotransferase): GLPK; glycerol-3-phosphate acyltransferase: GPAT; lysocardiolipin and lysophospholipid acyltransferase: LPAT; Diacylglycerol diphosphate phosphatase: LPP3; diacylglycerol O-acyltransferase 3: DGAT3; diacylglycerol O-acyltransferase 2: DGAT2; phospholipid: diacylglycerol acyltransferase: PDAT; sn-Glycerol 3-phosphate: G-3-P; lysophosphatidic acids: LPA; Phosphatidic acid: PA; Diacylglycerol: DAG; Triacylglycerol: TAG; phosphatidylcholine: PC; glycerophosphodiester phosphodiesterase: GDPD1; sn-Glycero-3-phosphoethanolamine: Glycerophosphoethanolamine; sn-Glycero-3-phosphocholine: Glycerophosphocholine; phosphatidylcholine diacylglycerol cholinephosphotransferase: ROD1, PDCT; lysophosphatidylcholine acyltransferase: LPCAT; free fatty acid: FFA; lysophosphatidylcholine: LPC. The vertically stacked blocks represent different genes encoding enzymes with this catalytic function, and the horizonal direction represents different stages.

Based on the gene annotation and FPKM analysis of *S. saponaria* kernel transcriptome at three critical stages, this study focused on elucidating the metabolic pathways associated with the biosynthesis of lipid accumulation in soapberry kernels. During the development of soapberry kernels, DEGs related to FA biosynthesis and elongation, UFA and TAG biosynthesis were identified, facilitating a more comprehensive understanding of the expression patterns of oil accumulation genes in *S. saponaria* kernels (Figure 4).

In the cytoplasm, acetyl-CoA carboxylase (ACCase) catalyzes the conversion of acetyl coenzyme A (acetyl-CoA) to malonyl-CoA, which is the first step in the FA biosynthesis pathway. In soapberry, the ACCase gene *CAC3* is responsible for this conversion. The gene exhibits the highest FPKM expression level during the S1 stage (70 DAF), and its expression is subsequently down-regulated in the S2 (100 DAF) and S3 (130 DAF) stages. Acetyl-CoA, derived from pyruvate, serves as the initial substrate for FA synthesis. The pyruvate dehydrogenase complex (PDC) catalyzes the conversion of pyruvate to acetyl-CoA. In soapberry, *PDC* shows the highest expression level in the S1 stage. Among FA synthase, acyl carrier protein (ACP)-S-malonyltransferase (FabD, *FabD*) and enoyl-ACP reductase I (FabI, *MOD1*) enzyme genes were up-regulated in the S2 period, and *FabD*, 3-oxoacyl-ACP synthase II (FabF, *KASI*), 3-hydroxyacyl-ACP dehydratase (FabZ, *FabZ*), 3-oxoacyl-ACP reductase (FabG, *FabG*) enzyme gene was down-regulated in the S3 stage (Figure 4). Most of the DEGs related to FA synthase exhibit higher FPKM values in S1 and S2 compared to S3. The high expression of *PDC* and *CAC3* in S1 indicated that the initiation of lipid biosynthesis provided sufficient malonyl-CoA for subsequent acyl extension. The synthesis of malonyl-CoA is a rate-limiting step in FA synthesis and plays a key role in its regulation. During the three stages of soapberry kernel development (S1, S2, and S3), the proportion of C18:1 increased sequentially from 30% in S1 to 54% in S2 and 61% in S3 (Figure 1E). The activity of FA synthase increased during the rapid oil accumulation phase (S2) of soapberry kernels, providing a substrate source for the synthesis of long-chain FAs (Figure 4). 

Fatty acyl-ACP thioesterase A (FATA) and Fatty acyl-ACP thioesterase B (FATB) have substrate preferences, and FATA is mainly responsible for catalyzing the formation of C18:1 from C18:1-ACP. FATB, like FATA, plays an important role in fatty acid biosynthesis, with multiple enzyme functional sites and a preference for converting fatty acyl CoA with carbon atoms less than or equal to 18 into corresponding free fatty acids. *FATA* and *FATB* expression were down-regulated in the S2 and S3 stages. After the completion of biosynthesis, the FA must undergo conversion into fatty acyl CoA for FA elongation or participation in glycerolipid metabolism. The pivotal role in this process is played by long-chain acyl-CoA synthetase (LACS). The key enzyme genes of *LACS1* and *LACS6* were down-regulated in the S2 stage, while *LACS7* was up-regulated in the S2 stage and then down-regulated in S3 (Figure 4), which provided a substrate source for TAG assembly.

The content of C18: 0 decreased in the S1, S2, and S3 period. The difference is that C18: 1 and C18: 3 continued to rise in S1, S2, S3 (Figure 1E). This phenomenon is attributed to acyl-ACP desaturase (SAD), ω-6 FA desaturase (FAD2), ω-3 FA desaturase (FAD3), very-long-chain 3-oxoacyl-CoA reductase (ECR), and very-long-chain enoyl-CoA reductase (KCR). SAD can desaturate C18:0-ACP to generate C18:1-ACP. The FPKM of *SAD* in S1 and S2 are higher in S3, and the trend of expression changes is consistent with the accumulation of C18:1 content. KCR and ECR are two important enzymes involved in the elongation of very-long-chain fatty acid carbon chains. They make significant contributions to the elongation of fatty acid carbon chains with carbon atoms greater than or equal to 16 in soapberry kernels, mainly participating in the elongation of C18:0-CoA carbon chains to form C20:0-CoA. FAD2 can desaturase the C18:1-PC form C18:2-PC. *FAD2* was down-regulated in S2 and S3, which provided another possibility for the accumulation of C18:1 (Figure 1E and Figure 4). FAD3 desaturates C18:2-PC to generate Linolenic-PC (C18:3-PC). Lysophosphatidylcholine acetyltransferase (LPCAT) is an important prerequisite for the formation of C18:2 and C18:3, as it converts C18:1-CoA into C18:1-PC, providing a reaction substrate for FAD2 (Figure 4).

### 2.8. Identification and Expression Profile of Key DEGs for TAG Assembly

Most FAs exist in the form of TAG or phosphoglycerides. Acyl glycerol is synthesized from two precursors, fatty acyl-CoA and glycerol-3-phosphate (G-3-P). Fatty acyl-CoA derived from the activation of FAs by acyl-CoA synthetase. G-3-P can be obtained from two sources. One source is dihydroxypyruvate, an intermediate in glycolysis, and the other source is glycerol degradation, which involves the phosphorylation of glycerol. TAG assembly takes place in the ER and occurs through both the acyl-CoA-dependent pathway (Kennedy pathway) and acyl-CoA-independent pathways. G-3-P and acyl-CoA considered as the main substrate of TAG [12,21,22,23].

Glycerol is converted to G-3-P under the catalysis of glycerol kinase (ATP: glycerol-3 phosphotransferase, GLPK). The primary source of glycerol relies on the catalytic activity of alcohol dehydrogenase (AKR) to produce D-glyceraldehyde. D-glyceraldehyde is derived from the conversion of D-glycerate by acetaldehyde dehydrogenase (ALDH). Another source of G-3-P is dihydroxypyruvate, which is generated through the catalysis of glycerin diphyate (sn-Glycero-3-phosphoethanolamine, sn-Glycero-3-phosphocholine) and H_2_O by glycerophosphodiester phosphodiesterase (*GDPD1*). The high expression of *AKR* and *ALDH* key enzyme genes in the S1 stage provides a source of G-3-P substrate for the rapid accumulation of oil in the S2 stage. *GDPD1* was continuously up-regulated in S2 and S3, while *GLPK* was up-regulated in S2 and down-regulated in S3. They work together continuously to provide G-3-P for TAG assembly. Glycerol-3-phosphate acyltransferase (GPAT) catalyzes G-3-P to generate lysophosphatidic acid (LPA) during the accumulation of soapberry oil. Multiple homologous DEGs (*GPAT1*, *GPAT8*, and *GPAT3*) are involved in this process. *GPAT1* and *GPAT8* showed the highest FPKM in the S1 period, while *GPAT3* exhibited the highest FPKM in the S3 period. Lysocardiolipin and lysophospholipid acyltransferase (LPAT) synthesize phosphatidic acid (PA) using lysophosphatidic acid (LPA) as a substrate. Through differential expression gene screening, we identified 5 LPATs (*LPAT3*, *LPAT4*, *LPAT5*, *LPAT7*, *PLSC*), and their FPKM values reached the highest level in the S1 stage. Diacylglycerol diphosphate phosphatase (LPP3) can hydrolyze 1,2-diacylglycerol 3-phosphate (Phosphatidate) into 1,2-diacyl-sn-glycerol (DAG) and phosphoric acid. The gene encoding the key enzymes in soapberry is *LPP3*, and its FPKM values tend to decrease first and then increase, reaching the highest level in the S3 period. Phospholipid: Diacylglycerol acyltransferase (PDAT), diacylglycerol O-acyltransferase 3 (DGAT3) and DGAT2 represent the final enzymatic steps in triglyceride (TAG) synthesis. PDAT converts diacylglycerol (DAG) and phospholipid into lysophospholipid (LP) and TAG, while DGAT3 and DGAT2 catalyzes the conversion of acyl-CoA and DAG to generate CoA and TAG. These had the highest FPKM values during the S1 stage, with a decrease in the FPKM value in S2 and then an increase in S3 (Figure 4).

### 2.9. qRT-PCR

**Figure 5:**
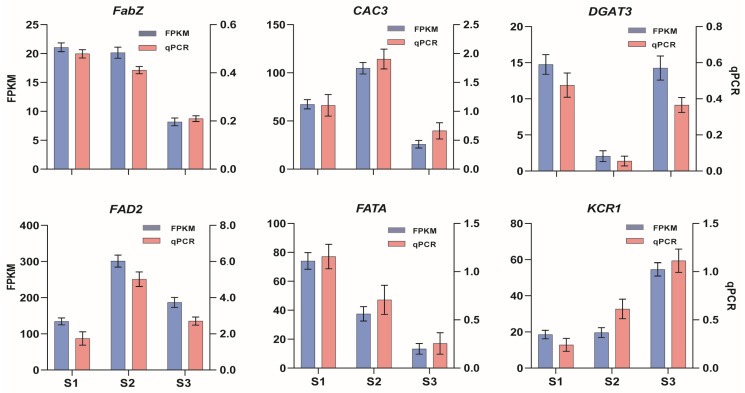
qPCR validation of six candidate genes associated with oil biosynthesis during *S. saponaria* kernel development.

To further validate the accuracy of transcriptome sequencing results for soapberry kernel, we performed qRT-PCR verification on six DEGs involved in lipid metabolism, namely *FabZ*, *CAC3*, *FATA*, *FAD2*, *DGAT3* and *KCR1*. The gene expression levels were assessed using qRT-PCR and found to be consistent with the RNA-seq results (Figure 5). Moreover, a significant correlation was observed between the relative expression levels of these six genes and the RNA-seq analysis outcomes. This further confirms the reliability of the soapberry kernels transcriptome sequencing expression profile and demonstrated the effectiveness of RNA-seq technology in detecting DEGs.

## 3. Discussion

### 3.1. S. saponaria Oil Content and Fatty Acid Content

Currently, there are increasing applications for oil from *S. saponaria*, but little is known about the molecular basis for its oil accumulation and the genes involved in oil biosynthesis. The release of the genome sequence of *S. saponaria* has helped to promote research in this field. We assembled the transcriptome of *S. saponaria* kernels and studied the dynamic gene expression profiles at three key developmental stages, as follows: 70 DAF, 100 DAF, and 130 DAF (Figure 3A). At the same time, the soapberry kernel oil contents and fatty acids at 11 different developmental stages of the fruit were studied, including C18:1, C18:3, C18:2, C20:0, C16:0, and C18:0. 

The oil content of *S. saponaria* kernels was the lowest, at 5%, in the early stage of oil accumulation (60 DAF), and the highest, at 31%, in the stable stage of oil accumulation (130 DAF), which is lower than the previous report of 39% [5]. The content of UFAs in the oil of mature soapberry fruit kernels was 86%, which is similar to the previously reported research results of 82% [5] and 85% [24]. The FA percentage was highest in C18:1 and C18:3, with an average proportion of 62% and 18% after fruit ripening (130–160 DAF), which is consistent with the research results of Chakraborty M. and Baruah D.C. (58% and 17%) [5]. However, this differs from the results of Shah. et al. [24] and Sengupta. et al. [25], with C18:2 and C22:0 being the second most abundant FAs. What can be confirmed is that the highest proportion of FAs was C18:1, which is consistent with our results, which were 59% and 63%, respectively. This may be due to differences in environmental conditions (climate, soil, light), varieties, cultivation management, and genetic background in different regions. The FA composition of the same family of yellow horn is also different from that of *S. saponaria*. The highest proportion of FA in yellow horn was C18:2 (42–54%) and C18:1 (22–28%) [26]. *Prunus pedunculata* [18] and *Armeniaca sibirica* [27] have the highest fatty acid content with a C18:1 ratio. Unlike soapberry, the second highest proportion of fatty acids in *Prunus pedunculata* [18] and *Armeniaca sibirica* [27] was C18:2, rather than C18:3. This is different than the oil content of different woody oil plants, and the FAs are also different, but there are certain similarities, such as the content of UFAs and the main FAs.

### 3.2. Fatty Acid Biosynthesis Key Enzyme Genes in S. saponaria Kernels

The biosynthesis of FAs and TAGs constitutes a pivotal aspect of lipid metabolism. The metabolic pathways governing each essential component are intricate processes involving multiple sequential steps and is regulated by multiple enzymes and genes. Previous studies have revealed differences in oil content, FA composition, and FA content ratio among different woody oil plants. These differences can be attributed to variations in the activity of key enzymes involved in lipid metabolism and the expression levels of corresponding key enzyme genes. ACCase plays a pivotal role in controlling FA biosynthesis as a key rate-determining step. However, attempts to increase seed oil production by up-regulating ACCase have yielded only modest success due to the complex regulation of ACCase activity, which involves factors such as light, phosphorylation, thioredoxin, PII protein, and product feedback control [28,29,30,31]. Acyl-ACP thioesterase (FAT) can be classified into two types, FATA and FATB, based on their amino acid sequences. These enzymes exhibit substrate preferences and selectivity. FATA tends to catalyze C18:1-ACP and C18:0-ACP, with a preference for C18:1-ACP, while FATB prefers to catalyze C16:0-ACP [32,33,34]. Both *FATA* and *FATB* were active during soapberry kernel development; however, in terms of gene expression FPKM value, FATA consistently exhibited higher values than FATB at all time points, similar to those observed in *Prunus pedunculata* [18] and *Prunus sibirica* [19]. 

The average percentages of C18:1 and C16:0 in mature soapberry fruit kernels were 62% and 6%, respectively. The proportion of C18:1 was significantly higher than that of C16:0, indicating consistency between the GC detection results of *S. saponaria* kernels and the transcriptome results. The elevated C18:1 content can be attributed not only to the higher activity of the FATA enzyme compared to FATB, but also to the conversion of C16:0-ACP into C18:0-ACP by the KASII, which provides ample C18:0-ACP substrates for SAD to catalyze the synthesis of C18:1-ACP. This may also explain why C18:1 constitutes the highest proportion of fatty acids in soapberry kernels. The key enzyme genes *LACS1* and *LACS6* were down-regulated in the S2 stage, while *LACS7* was up-regulated in the S2 stage and subsequently down-regulated in S3. This pattern aligns with the minor changes observed in the content of long-chain FAs after the S2 stage. The average proportion of C18:2 in soapberry kernels after fruit ripening was 6%, and the C18:3 was 18%, second only to C18:1. It was found that there were not only DEGs encoding *FAD2* but also DEGs encoding *FAD3* in transcriptome analysis of *S. saponaria* kernels. FAD3 can generate another double bond at the ∆^15cis^ position of C18:2-PC, resulting in C18:3-PC (C18:3^Δ9cis,12cis,15cis^) with three double bonds [17,35]. This indicates that C18:2-phosphatidylcholine (C18:2-PC) in the soapberry kernels can be derived from the desaturation of C18:1-PC, and C18:2-PC can further desaturate to generate C18:3-PC. This is same as the acyl-CoA-dependent pathway synthesis of C18:3 in flax [36] and *Camelina sativa* [37].

### 3.3. S. saponaria TAG Biosynthesis and Oil Accumulation

The synthesis of TAG takes place in the ER, and the pathway from G-3-P and acyl-CoA to TAG assembly consists of multiple enzyme-catalyzed reactions (Figure 4) [38]. Initially, GPAT transfers FA from acyl-CoA to the sn-1 hydroxyl of G-3-P, producing LPA. Subsequently, LPAT esterifies the second FA to generate PA. 

Then, diacylglycerol diphosphate phosphatase (LPP3) removes the sn-3 phosphate to yield DAG. DAG is crucial, as it can be utilized in the biosynthesis of membrane lipids, such as phosphatidylcholine (PC) and phosphatidylethanolamine, or in TAG production. Additionally, DAG used for TAG biosynthesis can also be obtained from PC [39]. In the final crucial step, there are two pathways for TAGs synthesis, as follows: the acyl-CoA-dependent pathway (Kennedy pathway) and the acyl-CoA-independent pathway, with the main difference lying in the conversion of DAG to TAG [21,22,23]. In the Kennedy pathway, DGAT transfers the fatty acyl group from acyl-CoA to the sn-3 position of DAG to synthesize TAG [40]. Another pathway involves PDAT transferring acyl groups from PC to DAG, or through DGTA generating one TAG and one monoacylglycerol (MAG) without relying on acyl-CoA [41]. During the development of the *S. saponaria* kernel, three DGATs (1 *DGAT2* and 2 *DGAT3* homologous genes) and three PDATs (3 *PDAT1* homologous genes) were identified. Throughout the maturation process of the *S. saponaria* kernel, the FPKM of DGAT is significantly higher than that of PDAT, and PDAT exhibited continuous down-regulation in S2 and S3 stages, indicating that DGAT is the main gene involved in TAG synthesis. DGAT serves as the sole rate-limiting enzyme in TAG synthesis in the Kennedy pathway. In soapberry kernels, two genes encoding type III DGAT (*SsDGAT3*) and one gene encoding type II DGAT (*SsDGAT2*) were identified. However, *DGAT2* is primarily engaged in the synthesis of FAs with fewer than 16 or more than 18 carbon atoms. The average content of C20:0 after fruit ripening in soapberry kernels was 6%, which is much lower than the total percentage of C16:0, C18:0, C18:1, C18:2, and C18:3. Therefore, it is inferred that DATG3 is the main enzyme gene for TAG synthesis in *S. saponaria* kernels [42,43,44,45,46].

## 4. Conclusions

The accumulation of oil in soapberry kernels can be categorized into the following three crucial periods: an initial slow accumulation period (60–90 DAF); a subsequent rapid accumulation period (90–130 DAF); and finally, a stable accumulation period (130–160 DAF). The oil content in *S. saponaria* kernels reached its lowest point at 5% at 60 DAF during the early stage of oil accumulation and peaked at 31% at 130 DAF during the stable period of oil accumulation. After 100 DAF, the fatty acid composition in soapberry kernels tends to stabilize. Following fruit ripening (130–160 DAF), the main fatty acids present were C18:1 and C18:3, accounting for an average proportion of 62% and 18%, respectively. The average percentage of UFAs and SFAs in the kernels was 86% and 14%, respectively. Based on the soapberry fresh weight of 100 fruits, the oil content in the kernels, and the dynamic fatty acids, the optimal harvest period for the fruit is 130 DAF, when it matures. The transcriptome analysis results showed that the DEGs involved in FA synthesis and TAG assembly were consistent with the expression patterns of all genes in the kernels, exhibiting high expression in the early stage of oil accumulation (70 DAF). Through dynamic changes in FA content and DEGs analysis, *FATA*, *KCR1*, *ECR*, *FAD2* and *FAD3* were candidate genes that contribute to a high proportion of C18:1 and C18:3 in *S. saponaria* kernels. Additionally, *DGATs* was the key candidate gene for TAG biosynthesis in soapberry kernels.

## 5. Materials and Methods 

### 5.1. Plant Material 

The *S. saponaria* fruits were collected in Hunan Soapberry Agroforestry Development Co., Ltd., Changde, Hunan, China (111.127309; 29.766501). Soapberry fruits at different developmental stages (10–160 DAF) were collected based on phenological observations. Three plants were selected for each collection period, and fruits were collected from each plant in the four cardinal directions (east, south, west, north). A portion of the kernels (1.0 g) was taken from each fruit, immediately placed in liquid nitrogen, and transported back to the laboratory for storage at −80 °C. This portion of the kernels was used for RNA extraction for RNA-Seq and qT-PCR analysis. The remaining kernels from the fruits were brought back to the laboratory and dried for determination of the oil content and fatty acid content.

### 5.2. Kernels Oil Extraction

The extraction of soapberry kernel oil was conducted following the Chinese National Standard In “Determination of Fat in Foods” (GB 5009.6-2016) method [47], which is also known as the Soxhlet extraction method. This process was repeated three times. The sample pretreatment and test procedures were conducted according to the method of Zhang et al. [48]. After each sample collection was completed, the soapberry fruits were promptly brought back to the laboratory and dried in a constant-temperature drying oven (101-1A, Beijing Kewei Yongxing Co., Ltd., Beijing, China) to remove moisture (85 °C, 12 h). The kernels were then ground into a fine powder using a grinder (DFY-500, Shanghai Xinnuo Instrument Group Co., Ltd., Shanghai, China). The oil content of the dry kernels was determined using an SZE-101 fat analyzer (Shanghai ShineJan Instruments, Shanghai, China). Prior to oil extraction, the soapberry kernel powder was dried in an oven until a constant weight was achieved (85 °C, 24 h). The weight of the kernel powder (approximately 3.0–4.0 g) was measured (M_0_). The extraction process was carried out using petroleum ether (99.7%, boiling range 30–60 °C) for 6–8 h. The residue remaining after oil extraction was dried in a 105 °C oven for over 2 h and weighed (M_1_). The total oil content of the kernels (w) was calculated using the following formula: W = (M_0_ − M_1_)/M_0_ × 100% (weight accurate to 0.0001 g).

### 5.3. Gas Chromatography Analysis of Fatty Acid Methyl Esters

The determination of FA methyl esters and content was conducted following the GB 5009.6-2016 method [47]. For the FA methyl ester analysis, a soapberry kernel oil sample of approximately 0.06 g was weighed and mixed with 4 mL of isooctane and 200 μL of a potassium hydroxide–methanol solution (2 mol/L). The mixture was thoroughly shaken for 60 s and allowed to settle for 30 min. Then, 1.0 g of anhydrous NaHSO_4_ was added, and the mixture was shaken for 30 s and allowed to settle for 10 min. The supernatant was collected and filtered using a 0.22 μm organic filter membrane for measurement. The FA content of soapberry was determined using gas chromatography (Shimadzu Nexis GC-2030: Shimadzu, Kyoto, Japan). The program settings for the GC analysis were conducted according to [12].

Equipped with an Agilent HP-88 quartz glass capillary column (0.25 mm × 100 m × 0.20 μm), the helium flow rate was set at 1.1 mL/min, and the split ratio was 1:100. The temperature program was set as follows: the initial temperature was 100 °C and maintained for 13 min, then increased from 100 °C to 180 °C at a rate of 10 °C/min, followed by a 6 min hold at 180 °C. Subsequently, the temperature was increased from 180 °C to 200 °C at a rate of 1 °C/min, held at 200 °C for 20 min, and finally increased from 200 °C to 230 °C at a rate of 4 °C/min, with a 10.5 min hold at 230 °C for detection. During detection, the separation was greater than 1.25, the inlet temperature was 270 °C, and the injection volume was 1.0 μL. The Supelco 37 Component FAME (fatty acid methyl ester) Mix (Supelco: CRM47885, Bellefonte, PA, USA) was used as the standard to identify fatty acid components, and the peak area was used to determine the relative content of fatty acids in the *S. saponaria* kernels.

### 5.4. RNA-Seq and Analysis

Total RNA from *S. saponaria* kernels was extracted using Trizol kit (Invitrogen, Carlsbad, CA, USA). RNA quality was assessed on an Agilent 2100 Bioanalyzer (Agilent Technologies, Palo Alto, CA, USA). After extracting total RNA, Oligo (dT) beads were used to enrich eukaryotic cell mRNA and remove rRNA to enrich prokaryotic mRNA. The enriched mRNA was then fragmented into short fragments using a fragmentation buffer and reverse-transcribed into cDNA using the NEBNext Ultra RNA Library Preparation Kit for Illumina (NEB #7530, New England Biolabs, Ipswich, MA, USA) and sequencing.

The raw sequencing data underwent quality control using fastp, where low-quality data was filtered out to obtain high-quality data. The sequence assembly was performed using Trinity software [49,50]. For functional annotation, the published Genome of *S. saponaria* [1] was used as a reference, and the clean data was matched with the KEGG public database. Quantitative analysis was conducted using RSEM (RNA-Seq by Expectation Maximization) [51].

The DESeq2 [52] software was utilized for conducting gene differential expression analysis, and it was determined that the screening difference factor exceeded 2. The expression analysis employed an FPKM value and FDR value less than 0.05, with the screening parameters being FDR < 0.05 or |log2FC| > log2(2). Based on these criteria, differential expression analysis between different developmental stages of kernels was performed, and the key enzyme genes involved in the lipid metabolism of the soapberry kernels were compared.

### 5.5. qT-PCR Validation of RNA-Seq

Five key genes, *FabZ*, *CAC3*, *PDAT*, *FATA*, and *DGAT3*, which are related to the lipid biosynthetic pathway, were selected for validation (Table S4). *GAPCP1* (enzyme protein) was used as the internal reference gene for quantification. The primers for these genes were designed using Premier 5.0 software (Premier Biosoft International, Palo Alto, CA, USA). The qT-PCR reaction conditions for all samples included an initial denaturation step of 3 min at 95 °C, followed by 40 cycles of amplification. Each cycle consisted of a 15 s denaturation step at 95 °C, a 15 s annealing step, and a 30 s extension step at 60 °C. Finally, there was a 15 s denaturation step at 95 °C. Each reaction was repeated three times. The relative expression level of each single gene was determined using the comparative cycle threshold (ΔΔCt) method [53].

## Figures and Tables

**Table 1:** Statistical overview of soapberry seed kernel transcriptome assembly data.

Category	Value
RawData (bp)	61,500,521,100
RawDatas	410,003,474
CleanData (bp)	60,716,969,523
CleanDatas	407,463,420
CleanData GC (%)	45.91%
CleanData Q20 (%)	97.51%
CleanData Q30 (%)	93.01%

## Data Availability

The data presented in this study are available within the article and Supplementary Materials. The *S. saponaria* kernels transcriptome raw data can be obtained from the China National Center for Bioinformation (https://ngdc.cncb.ac.cn), GSA number is CRA014727.

## Supplementary Materials

The following supporting information can be downloaded at: https://www.mdpi.com/article/10.3390/plants13131781/s1, Table S1: The comparison of raw and filtered transcriptome assembly data from *S. saponaria* kernels. Table S2: The transcriptome sequence alignment and gene statistics of *S. saponaria* kernels. Table S3: The numbers of the DEGs in 13 lipid metabolism KEGG pathways. Table S4: The qT-PCR gene primers sequence for RNA-seq validation in *S. saponaria* kernels. Figure S1: The GO and KEGG annotations of the transcriptome of *S. saponaria* kernels.
