# Supplementary figures and images for: The Biosynthesis Pattern and Transcriptome Analysis of Sapindus saponaria Oil
Source: Plants (Basel). 2024 Jun 27;13(13):1781. doi: 10.3390/plants13131781 (PMC11244568; doi:10.3390/plants13131781)

A

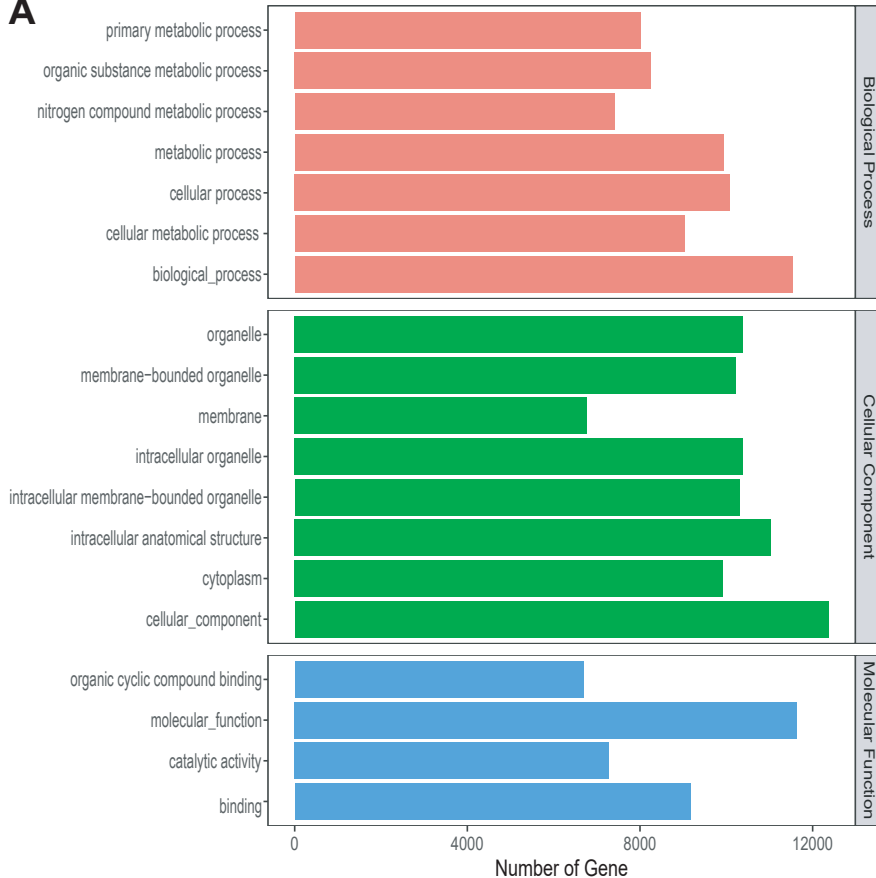

B

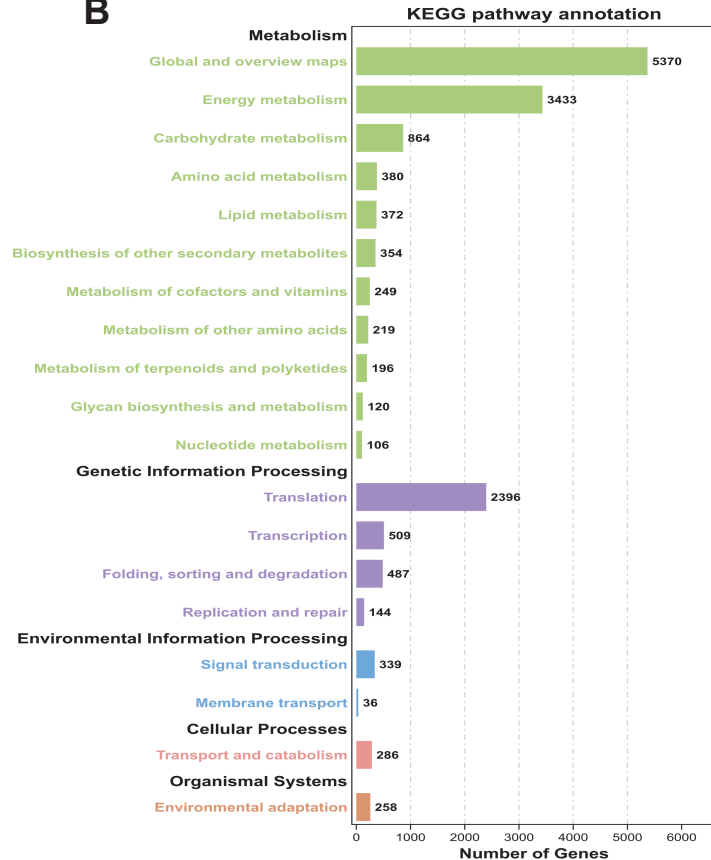

Supplement: Supplementary file 1 [file plants-13-01781-s001.zip › Supplement/Figure S1.pdf]
